# What does validation of cases in electronic record databases mean? The potential contribution of free text†

**DOI:** 10.1002/pds.2086

**Published:** 2011-01-18

**Authors:** Amanda Nicholson, Anne Rosemary Tate, Rob Koeling, Jackie A Cassell

**Affiliations:** 1 Division of Primary Care and Public Health, Brighton & Sussex Medical School FalmerBrighton, UK; 2 Natural Language and Computational Linguistics, School of Informatics, University of SussexFalmer, Brighton, UK

**Keywords:** electronic health records, free text, validation, natural language processing

## Abstract

Electronic health records are increasingly used for research. The definition of cases or endpoints often relies on the use of coded diagnostic data, using a pre-selected group of codes. Validation of these cases, as ‘true’ cases of the disease, is crucial. There are, however, ambiguities in what is meant by validation in the context of electronic records. Validation usually implies comparison of a definition against a gold standard of diagnosis and the ability to identify false negatives (‘true’ cases which were not detected) as well as false positives (detected cases which did not have the condition). We argue that two separate concepts of validation are often conflated in existing studies. Firstly, whether the GP thought the patient was suffering from a particular condition (which we term confirmation or internal validation) and secondly, whether the patient really had the condition (external validation). Few studies have the ability to detect false negatives who have not received a diagnostic code. Natural language processing is likely to open up the use of free text within the electronic record which will facilitate both the validation of the coded diagnosis and searching for false negatives. Copyright © 2011 John Wiley & Sons, Ltd.

Electronic health records (EHRs) offer great potential for research, enabling the rapid identification of patients for inclusion in intervention or observational studies. As their use becomes more widespread, it is important to understand the structure of the data that constitute these records. Primary care records in the UK have been computerised for several decades and in the UK electronic records are almost universal in GP practices. Several anonymised databases of primary care records exist which have been used extensively in research studies,^1^ including the General Practice Research Database (GPRD – http://www.gprd.com). EHRs also exist in secondary care settings and the data are then collated for various administrative or research purposes such as national disease registers or Hospital Episode Statistics (HES).

EHR systems use a combination of structured coded data and unstructured free text fields. The balance between these two components varies across different record systems with some EHRs consisting entirely of coded data. In some systems, such as HES in the UK, professional coders enter the codes based on the clinical records but in others, such as primary care, the code is entered by the clinician as part of routine care. In primary care systems, where text and codes are entered during clinical care, the factors determining whether information is entered as text or code are poorly understood. Research studies find it difficult to access and use large amounts of free text – due to issues of confidentiality, costs of anonymisation and the need to structure/code the information contained. Hence nearly all studies that use the GPRD (or most other electronic record systems) rely on coded diagnoses to identify cases, and related validation studies attempt to show whether cases with diagnostic codes do indeed have that condition. ‘Validation’ is often reported as a quality marker both of the results of the research and of the records used. Two recent papers have reviewed the validation of diagnoses within the GPRD.^2^,^3^ They have provided an excellent summary of the types of studies undertaken and shown that most (90%) coded diagnoses, from a range of conditions, are ‘validated’. There are, however, currently systematic ambiguities in how the term validation is currently used in this field compared to other diagnostic contexts.

In this paper, we address three issues concerning the use of EHRs in research. First, we discuss what validation means in the context of EHR research and suggest that there are two distinct types of validation – internal and external. We then go on to explore the widespread failure in EHR research to address the question of false negatives, that is cases of the disease who have not received a diagnostic code. We argue that these should be identified as far as possible in any validation study. Finally, we discuss the relation of code-choice to validation, arguing that sensitivity analyses to investigate the impact of code choice on study results should become standard practice. In conclusion, we suggest that computational techniques, such as natural language processing (NLP) which access free text, have the potential to tackle these challenges.

## Confirmation or validation?

There is ambiguity about what is meant by validation. Two related but distinct concepts are being conflated in the existing validation studies. The accuracy of a diagnostic code within an electronic record depends on two steps: whether the code accurately reflects the practitioner's opinion and whether that diagnosis was correct.

1. *Did the GP think that the patient had this condition? – confirmation or internal validation*. Sometimes a tentative diagnosis is coded then subsequently excluded, but the code remains on the record. Occasionally, a code may be entered in error and not corrected. Without further information it is unclear whether the code actually reflects the overall content of the records. The majority of existing validation studies address this question using additional information from the practice, either using the additional data in the EHR in the form of a diagnostic algorithm or through questionnaires or record request to the GP. We would suggest that this process is correctly considered as confirmation of the code or internal validation rather than any external validation of the diagnosis. It is testing whether the code represents the GP record accurately. If a primary care-based diagnosis only is required for the research study, then such confirmation/internal validation from practice records is sufficient. In the past this has involved obtaining paper records but as Herrett *et al*.^2^ discuss this leads to a potential bias as only a (possibly non-representative) proportion of practices take part in such additional studies. We would question the need for such contact. Paper records are becoming less common in primary care and in many cases the electronic record, including free text, is considered the complete legal record.
2. *Was the GP correct? – external validation*. This is a more classic validation of the diagnosis against some gold standard, the form of which will vary according to condition. For some, such as myocardial infarction, this may involve formal diagnostic criteria or need linkage to other data such as HES or disease registers such as the cancer registries or Myocardial Ischaemia National Audit Project (MINAP http://www.rcplondon.ac.uk/clinical-standards/organisation/partnership/Pages/MINAP-.aspx). More often a hospital diagnosis reporting histology or the opinion of a specialist will suffice. Since the recording of information received from specialists or secondary care in GP databases is not standardised and may often not be coded but entered into free text, methods which allow text to be searched will facilitate such validation. We term this external validation as it uses information which has been directly or indirectly sourced from outside the GP practice.

The need for internal or external validation will therefore depend on the clinical condition, and the nature of the research question. For example, studies examining the management of conditions wholly contained within primary care can use confirmation or internal validation, since the focus is what the GP did once she/he had made the diagnosis. However, incidence studies for complex conditions managed in collaboration with secondary care may require external validation.

## Finding false negatives

A well-recognised weakness of existing validation/confirmation studies is that with a few exceptions^4^,^5^ they do not consider the cases which have been missed by relying on coded data, i.e. patients with the condition who do not have a diagnostic code. Any missed cases will consist both of cases where the GP did not make the diagnosis and diagnosed but uncoded cases. Identification of the undiagnosed cases will be difficult as it would require complete coding of symptoms and signs. Relevant diagnostic tests may not have been performed if the diagnosis was not considered.

We will, therefore, focus on the more tractable issue of diagnosed but uncoded cases. Here the GP has made the diagnosis but did not code it. The balance between the coding of diagnosis versus symptoms or signs is poorly understood and is likely to be related to decision-making and certainty in diagnosis.^6^ There is evidence that this balance may change over time, for example that depressive illness has been more likely to be coded as symptoms than as a diagnosis in recent years.^7^ Potentially stigmatising diagnoses may be more likely to be put in text only so that it does not occur in summary records. It has been estimated that only 50% of HIV positive patients have their diagnosis coded in their primary care records.^8^ It is not known how many of those without a code had the diagnosis recorded in text or whether the GP was unaware of the diagnosis. More research is needed to understand how clinicians use diagnostic codes.

At present, the extent of cases missing in an electronic record database is usually estimated by comparison of rates obtained from within the database with those from external sources.^9^ Unless heroic attempts are made to review thousands of case records by hand it is difficult to identify individual diagnosed but uncoded cases at present. There are resource implications for this labour-intensive work in addition to important issues about anonymisation and confidentiality.

## Bias due to variations in code-lists

The process of drawing up code-lists to identify all patients with a given clinical condition is a critical step in EHR studies. Multiple code-lists may be required within one study for many different conditions such as co-variates and confounders as well as disease endpoints. But the process of preparing such code-lists is far from straightforward, and lacks rigour. The same clinical condition can be described using many different codes. A patient with a given clinical condition might receive one of several possible diagnostic codes as well as, or instead of, one or more codes describing symptoms or investigations. This flexibility in the coding structure facilitates the clinical use of these codes, minimising the time spent searching for codes by practitioners. However, this multitude of codes for a given condition presents a challenge when data need to be aggregated.

The selection of codes used to identify patients with a condition will vary according to the particular research question to be answered, reflecting in part the degree of certainty of diagnosis required. Sometimes it may be important to identify all possible cases but in other studies the population may be restricted to cases where the diagnosis is more certain. This variability in code-lists may have major implications not only for the results of any confirmation studies but potentially for results of all studies using EHR. Herrett highlights three studies where different subsets of code-lists were used in sensitivity analyses as a form of validation.^2^ Differences in code-lists largely accounts for variation of sevenfold in estimates of incidence of rheumatoid arthritis.^10^,^11^ Authors have begun to examine the effect of code-list variation on study results^12^,^13^ but this is an area that needs further work. In our experience of looking at the management of pelvic inflammatory disease in primary care, codes classed as probable or possible had implications for the estimates of the care received.^14^ Cases with possible codes were less likely to receive recommended treatments, reflecting perhaps diagnostic uncertainty, and were excluded from the final analyses (unpublished data, details available from author).

## Future directions – natural language processing as a tool for EHR research

Given the challenges inherent in attempting confirmation and validation through free text, and the potential for extensive bias due to code-list choice, what else should we do to make EHR research more robust? Fortunately, natural language processing (NLP), a branch of computational linguistics, has the potential to transform the availability of free text for analysis.^15^ In NLP, machine learning techniques can be used to train algorithms to extract textual information that represents a code or concept, for example to find all the different ways that a diagnosis of rheumatoid arthritis might be expressed in free text. In this way, structured data can be derived from free text. Such automatic processing of text using NLP algorithms might facilitate searching of free text in, for example, primary care records. This could assist in internal and external validation by finding diagnoses in GP entered text or in letters or discharge summaries from secondary care. NLP might also help in the identification of false negatives where a diagnosis has been recorded only in text. When that is possible, variations in code-lists may become less important but, for now, it is important for researchers to explore the impact that code choice is having on their results by including sensitivity analyses.

**KEY POINTS**

- Most studies using electronic health records rely on coded data only.
- When assessing the validity of these codes, it is important to separate the concepts of internal validation (does the code reflect the practitioner's diagnosis?) and external validation (is this diagnosis correct?).
- Existing validation studies are often unable to look for false negatives, diagnosed cases which have not been coded.
- Developments in natural language processing may enable the more widespread use of the free text contained in electronic records.
- In the meantime, the impact of code-list choice on study results should be explored in sensitivity analyses.

There is of course potential for new errors to be introduced by any automated processing of text. For example, codes might be derived from text which describes a suspected or possible rather than certain diagnosis. NLP algorithms allow for inclusion of negation and other measures of uncertainty. Such uncertainty might be found within the text itself or could be derived from the context of the data, such as a GP entry compared to a more formal letter from secondary care. GPs vary in the extent to which free text is used and this variation will also affect the results of NLP.

The potential errors introduced into research results by using only coded data in electronic records have not yet been quantified. The PREP project (http://www.informatics.sussex.ac.uk/research/projects/PREP/1.htm) has been funded by the Wellcome Trust to explore the extent to which accessing the free text in GP records affects the results of research. In particular, we are asking whether information from free text changes incidence estimates for rheumatoid arthritis or estimates of the delay between first presentation and diagnosis of ovarian cancer. We are developing methodologies to facilitate this access to text, including techniques for anonymisation and NLP to produce structured data in the form of additional codes derived from text. As part of this development, we will assess the accuracy of the data produced by NLP. Complementary, field studies exploring the factors influencing data entry in GP surgeries will use a human computer interaction approach to increase our understanding of the balance between coded and unstructured data.

We recommend that free text is considered as an integral part of the electronic record and wherever possible is included in research studies, so that its contribution can continue to be assessed. Both internal and external validation require free text information and technological advances in free text processing mean that we may be within sight of automated internal and external validation, including searching for false negatives. The impact of code choice on study results should, in the meantime, be routinely investigated by the inclusion of sensitivity analyses.

## CONFLICT OF INTEREST

The authors declare no conflict of interest.
